# First reported surgical explantation of infected thoracic branched endograft requiring complex arch and descending thoracic aortic repair

**DOI:** 10.1093/icvts/ivaf073

**Published:** 2025-03-22

**Authors:** Michael A Catalano, Cecillia Chin, Nimesh D Desai, Kendall M Lawrence

**Affiliations:** Division of Cardiac Surgery, Department of Surgery, Hospital of the University of Pennsylvania, Philadelphia, PA, USA; Division of Cardiac Surgery, Department of Surgery, Hospital of the University of Pennsylvania, Philadelphia, PA, USA; Division of Cardiac Surgery, Department of Surgery, Hospital of the University of Pennsylvania, Philadelphia, PA, USA; Division of Cardiac Surgery, Department of Surgery, Hospital of the University of Pennsylvania, Philadelphia, PA, USA

**Keywords:** endograft explant, thoracoabdominal repair, aortic surgery

## Abstract

We present a 51-year-old woman with thoracic aortitis and rapidly enlarging descending thoracic aortic aneurysm in the setting of *Clostridium septicum* bacteraemia. After antibiotic treatment, she underwent left carotid–subclavian transposition and endovascular repair with thoracic branched endograft (TBE) with left carotid artery coverage. Three months postoperatively, she developed endograft infection, with a rapidly enlarging pseudoaneurysm at the proximal landing zone. She underwent TBE explant and descending thoracic aortic reconstruction. This report illustrates a rare complication and the first described surgical TBE explantation.

## CASE REPORT

A 51-year-old female with autoimmune disease characterized by migratory arthritis with positive antinuclear antibody and anti-dsDNA, on prednisone and hydroxychloroquine, presented with chest discomfort. She was found to have *Clostridium septicum* bacteraemia; computed tomography angiography (CTA) revealed a severely inflamed aortic arch of normal diameter, suggestive of aortitis. She was discharged on 6 weeks of intravenous piperacillin–tazobactam.

During treatment, she experienced persistent chest pain. Follow-up CTA showed growth of the distal arch to 3.5 cm. Based on aortic diameter and the objective of completing antibiotics, conservative management was continued. However, she returned with hypertensive emergency and recurrent chest pain. Blood cultures were negative, but repeat CTA revealed growth of her distal arch to 4.8 cm. Given the rapid growth, she was admitted for urgent intervention. She initially underwent left carotid–subclavian transposition to facilitate thoracic branched endograft (TBE) landing in zone 2 with coverage of the left carotid artery and side-branch to the left subclavian artery. This was performed 3 days following carotid–subclavian transposition. Her postoperative course was uncomplicated. She was discharged on aspirin, antibiotics, hydroxychloroquine and prednisone.

**Figure 1:**
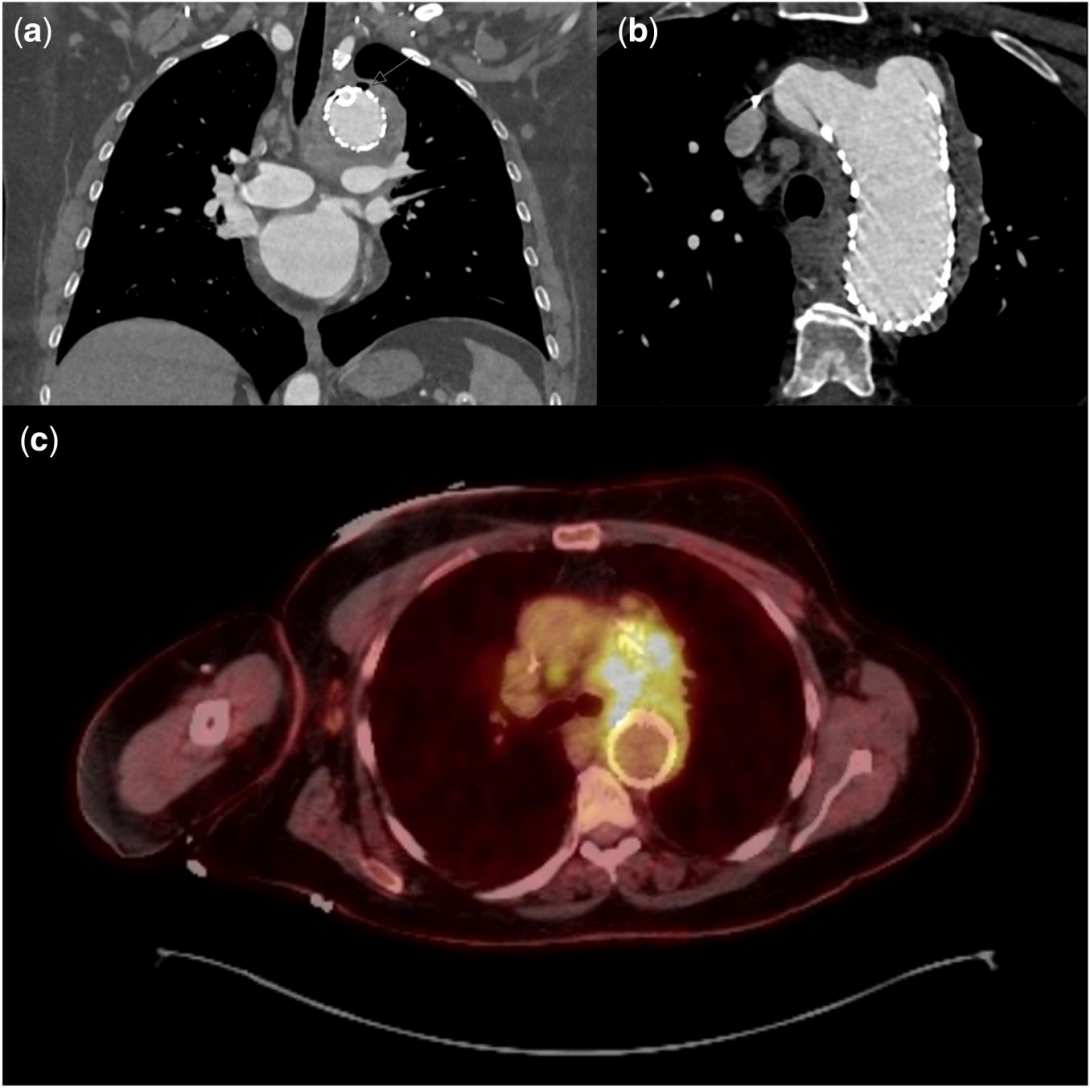
(a, b) CTA 2 months following TBE, demonstrating gas external to endograft with large pseudoaneurysm secondary to type 1a endoleak. (c) PET demonstrating significant FDG uptake at stented arch aneurysm. TBE: thoracic branched endograft

She returned with chest pain within the first month, but CTA revealed a well-positioned graft and stable aortic diameters. However, 1 month postoperatively, CTA revealed gas surrounding the graft at the aortic arch, related to inflammatory versus residual infectious aortitis. While blood cultures remained negative, she was treated with IV antibiotics given her chronically immunosuppressed state and the suspicion of infection. Three weeks later, imaging revealed a 4 cm saccular pseudoaneurysm at the proximal graft with persistent periaortic fluid and gas (Fig. 1). She was transferred to our institution for surgical explantation.

**Figure 2:**
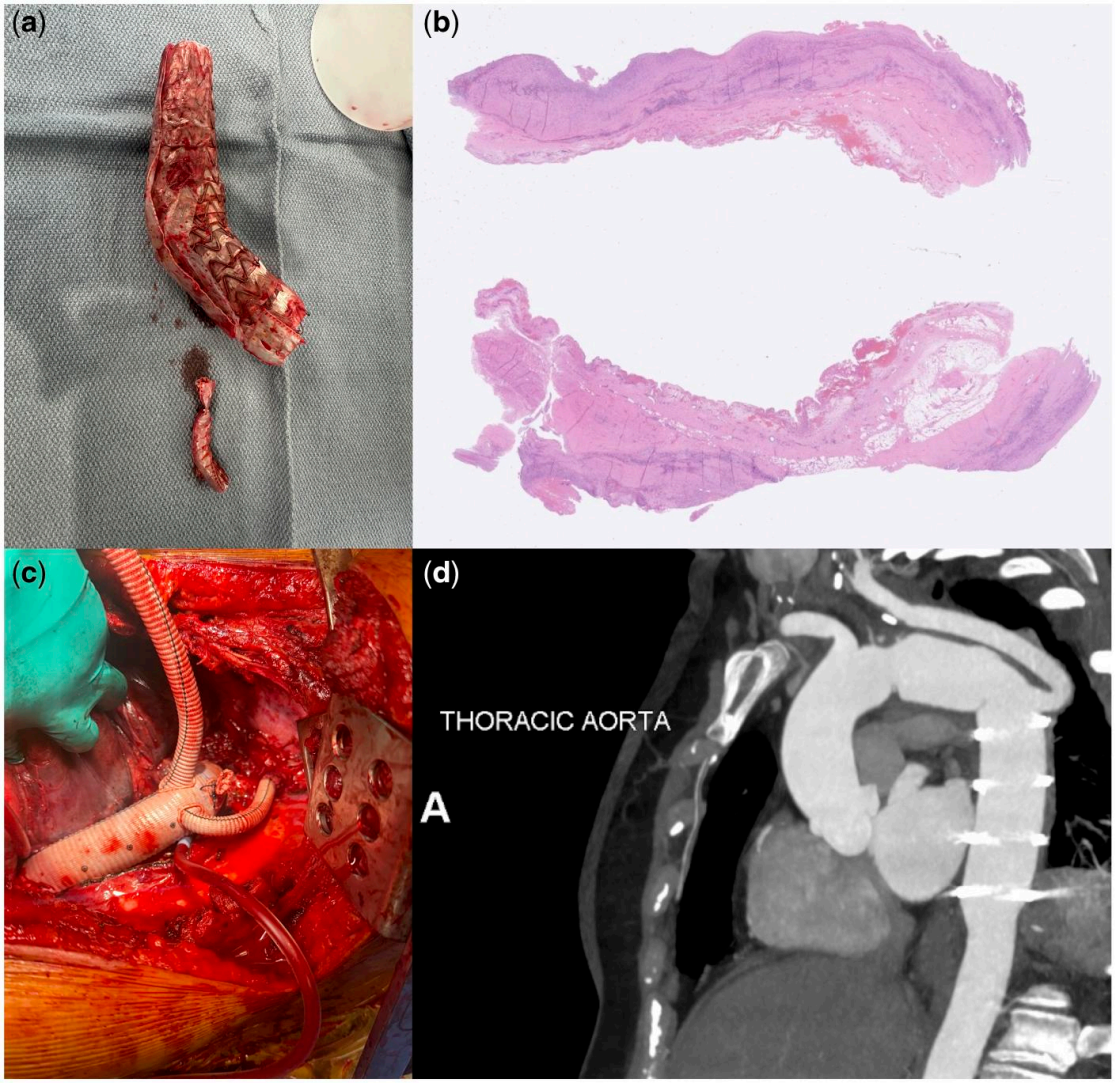
(a) Inflamed, explanted TBE; (b) pathologic assessment of resected aorta revealed marked acute and chronic inflammation and granulation tissue; (c, d) intraoperative photo and 2-month postoperative CTA demonstrating DTA and distal arch reconstruction. DTA: descending thoracic aortic; TBE: thoracic branched endograft

Following placement of a lumbar drain, the patient underwent left posterolateral thoracotomy and was cannulated for cardiopulmonary bypass via the descending thoracic aorta and the femoral vein. She was cooled to 18°C; upon electroencephalogram (EEG) silence, circulatory arrest with total body retrograde perfusion was initiated. The infected graft was explanted; the boggy, inflamed aorta was resected (Fig. 2). Proximal anastomosis was completed in zone 2, just distal to the ligated carotid orifice, with a 26-mm graft soaked in rifampin. Upper body reperfusion was re-initiated, and distal anastomosis was completed at the level of the diaphragm. During rewarming and full-body reperfusion, the subclavian artery was anastomosed to the 8-mm limb of the graft.

The patient tolerated the procedure well. Although tissue cultures remained negative, marked acute and chronic inflammation was noted in resected aortic tissue (Fig. 2). She was extubated on postoperative day 1, had lumbar drain removed on postoperative day 4 and was discharged on postoperative day 15 to rehabilitation with 6 weeks of antibiotics. She continues to do well 8 months postoperatively, with stable appearance of descending thoracic aortic graft on surveillance imaging (Fig. 2); cultures remain negative. She will continue to undergo close multidisciplinary clinical and radiographic surveillance of the repaired and native aortic segments.

The patient provided explicit verbal consent for publication of this report, obtained via phone by the attending surgeon.

## COMMENT

Thoracic aortic endograft infection, though rare, carries a mortality rate of over 70% in some series [1]. As the utilization of thoracic endografts increases, the incidence of infection may rise. Further, as branched endografts facilitate treatment of ascending and arch pathology, the complexity of surgical management of infected endografts will increase [2]. Surgical explantation remains the standard treatment, particularly in patients with anatomic complications, including pseudoaneurysm and fistulization [1, 3, 4]. To our knowledge, this is the first described surgical explantation of a branched endograft located in zone 2.

This case highlights the need for surgical intervention when conservative management fails. This patient was initially managed conservatively, given overall stability and negative cultures. Based on existing data, this is a reasonable approach—Moulakakis *et al*. [1] identified in-hospital mortality of 42.0% in conservative management of infected thoracic endografts, compared to 36.6% with surgical explantation. However, in this case, surgery was required due to a rapidly progressing pseudoaneurysm.

Surgical approaches to endograft infections vary; in this case, open resection with *in-situ* reconstruction was possible because of accessible healthy tissue. While extra-anatomic bypasses are used when complete graft excision is difficult, this patient’s anatomy allowed for resection and reconstruction via a single thoracotomy incision [5]. Adequate resection of all infected-appearing tissue was key, given the significant morbidity associated with recurrent graft infection. Additionally, in this case, particular care was required due to the prior carotid–subclavian transposition, routing antegrade flow to the left carotid via the stented left subclavian artery. Fortunately, healthy-appearing tissue was identified at each anastomotic site. The patient remains with appropriate antegrade flow through both vessels, without signs of steal.

## CONCLUSION

This report illustrates an early infectious complication of thoracic branched endograft placement and the first described successful repair. The clinical trajectory highlights the aggressive nature of inflammatory aortic pathology, emphasizing the importance of imaging surveillance to detect disease progression. Our surgical approach highlights a technique to safely explant and reconstruct a complex branched endograft. While surgical explantation and *in-situ* reconstruction were appropriate in this patient, further assessment of the short- and long-term outcomes of various approaches to infected aortic arch endografts is warranted. Further, as the prevalence of endovascular arch reconstruction increases, complex endograft explantation may soon become a more commonly encountered surgical endeavour.

## Data Availability

The data underlying this article are available in the article; any further request for data regarding this case will be shared on reasonable request to the corresponding author.

## Notes

Presented at the 2024 STS-ASCVTS Aortic Summit, Tokyo, Japan, November 15, 2024.
